# Investigating the Mediating Role of Mental Disorders in the Relationship Between Early Sexual Intercourse and Intentional Self‐Harm: A Two‐Step and Multivariable Mendelian Randomization Study

**DOI:** 10.1002/brb3.70124

**Published:** 2024-12-03

**Authors:** Dameng Dong, Saboor Saeed, Rucheng Chen, An Chen, Weijun Zheng

**Affiliations:** ^1^ School of Public Health Zhejiang Chinese Medical University Hangzhou Zhejiang China; ^2^ School of Medicine Zhejiang University Hangzhou Zhejiang China; ^3^ Department of Public Health, Faculty of Medicine University of Helsinki Helsinki Finland

**Keywords:** early sexual intercourse, intentional self‐harm, Mendelian randomization, mental disorders

## Abstract

**Background:**

Epidemiological studies have established an association between early sexual intercourse and intentional self‐harm. However, the causal mechanisms, particularly the role of mental health disorders, remain elusive.

**Methods:**

In this Mendelian randomization (MR) study, we leveraged genome‐wide association study data from multiple sources. Age at first sexual intercourse (AFSI) statistics were derived from the UK Biobank, encompassing up to 406,457 participants of European ancestry. Intentional self‐harm (ISH) genome‐wide association study (GWAS) data were obtained from the FinnGen Biobank, comprising 218,792 European‐descent individuals. We investigated the causal relationship between AFSI and ISH and quantified the mediating effects of major depressive disorder (MDD; *n* = 173,005), schizophrenia (SCZ; *n* = 127,906), and attention deficit hyperactivity disorder (ADHD; *n* = 55,374). Both two‐step and multivariable MR approaches were employed to estimate the mediation effects of these psychiatric disorders.

**Results:**

The *F*‐statistics of all retained SNPs are over 10, indicating our study has no weak instrument bias. The odds ratio (OR) indicated that early sexual intercourse substantially increases the risk of ISH (IVW: OR = 0.60, 95% CI = 0.54–0.66, *p* < 0.05). Specifically, MDD was found to mediate 31% of this effect and SCZ 12%, collectively accounting for 46% of the total effect.

**Conclusions:**

Early sexual intercourse is associated with an increased risk of intentional self‐harm, potentially mediated by psychiatric disorders. These findings emphasize the need for mental health screening and early intervention in adolescents with early sexual debuts.

## Graphical abstract

Mendelian randomization is a statistical method that relies on genetic variations as instrumental variables to explore causal associations between exposure and outcome. Utilizing a two‐step and multivariable Mendelian randomization analysis, it was concluded that major depressive disorder and schizophrenia partially mediate the relationship between early sexual intercourse and intentional self‐harm.

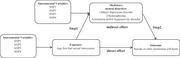

## 1 Introduction

Suicide and self‐harm, recognized as major health and societal issues, continue to occur all over the world and have a profound impact on human lives and deaths (Knipe et al. 2022; Jakobsen et al. 2023; World Health Organization 2019). Data from the World Health Organization reveal that suicide claims nearly 700,000 lives annually, remaining a leading cause of death among 15–29‐year‐olds worldwide (Hawton 2014). Self‐harm is believed to be even more common, affecting an estimated 14.6 million individuals annually (Global Burden of Disease Collaborative Network 2019), with a particularly high prevalence (around 17%) observed among adolescents (Gillies et al. 2018).

ISH is defined by the International Classification of Diseases, Tenth Revision (ICD‐10) (Randall et al. 2017) as any deliberate, self‐inflicted poisoning or injury, regardless of the intended outcome or lethality. While self‐harm is a broad category that includes any act of self‐inflicted injury, intentional self‐harm specifically refers to those actions where the individual clearly intends to cause harm to themselves. It encompasses a range of behaviors, including suicide deaths, suicide attempts, and non‐suicidal self‐injury (Turner et al. 2018). In the United States alone, these behaviors result in over 40,000 deaths and nearly 500,000 emergency department visits each year (Kolbe, Kann, and Collins 1993). Individuals who engage in ISH exhibit significantly compromised physical health and life expectancy compared to the general population (Bergen et al. 2012). Therefore, it is crucial to understand the causal factors to develop effective prevention strategies.

Early sexual intercourse (ESI) has been associated with various adverse health outcomes, including increased risks of sexually transmitted infections, substance use, and unintended pregnancies (Reis et al. 2023; Shu et al. 2016). Recent studies indicate a concerning trend of decreasing age at first sexual intercourse (AFSI), with 14.2% of adolescents aged 12–15 reporting sexual activity (Kushal et al. 2022). The relationship between the initiation of sexual activity and deliberate self‐harm has been reported as a significant association (Patton et al. 2007). Prior Mendelian randomization (MR) studies have substantiated the causal effect of the age of first sexual intercourse on the likelihood of self‐harm and suicide attempts (Lu et al. 2023). However, the intricate association and mechanisms underlying this relationship between the AFSI and ISH require further elucidation.

Observational studies have shown that individuals initiating sexual intercourse between ages 12–14 face elevated risks of mental disorders and suicidal tendencies (Mota et al. 2010). Common mental disorders such as major depressive disorder (MDD), schizophrenia (SCZ), and attention deficit hyperactivity disorder (ADHD) are frequently observed in cases of early sexual activity and ISH (McEvoy et al. 2023; Ramrakha et al. 2000). Previous MR studies have demonstrated the causal effects of early sexual initiation on MDD (Lu et al. 2023), SCZ (Yu et al. 2023), and ADHD (Soler Artigas et al. 2023) and the subsequent impact of these disorders on self‐harm (Lim et al. 2020). These findings suggest that mental health issues may partially mediate the effect of AFSI on ISH, but none of them have quantified the mediation effect.

To address this gap, our study utilized MR (Davey Smith and Hemani 2014), which had served as an alternative approach to large‐scale epidemiological cohort studies for assessing causal relationships between exposures and outcomes. MR relies on genetic variants as instrumental variables (IVs) to examine associations between phenotypic exposure and outcome (Li et al. 2022). MR's strength lies in its ability to minimize confounding factors and reverse causality. This approach provides a more definitive exploration of the connections between AFSI, mental disorders, and ISH (Davies, Holmes, and Davey Smith 2018). Recently, the MR method has been applied in investigating mediating pathways (Carter et al. 2021). In this study using the MR technique, we aimed to explore the extent to which mental disorders (MDD, SCZ, and ADHD) mediate the relationship between AFSI and ISH. The findings of this study have the potential to significantly impact our understanding of the complex interplay between early sexual activity, mental health, and self‐harm behaviors. By elucidating these relationships, we can contribute to the development of more targeted and effective interventions for preventing ISH, particularly among vulnerable adolescent populations.

Furthermore, our research has important implications for public health policy and clinical practice. Understanding the mediating role of mental disorders in the relationship between AFSI and ISH can inform the design of comprehensive prevention strategies that address both sexual health and mental well‐being (Grasdalsmoen et al. 2020; Lovero et al. 2023). This knowledge may also help healthcare providers identify high‐risk individuals earlier and implement timely interventions, potentially reducing the incidence of self‐harm and its associated morbidity and mortality (Hua et al. 2024).

Our study quantifies mental disorders' mediation between first sexual intercourse age and self‐harm, addressing a key literature gap. These findings may inform targeted risk assessment and intervention strategies for at‐risk individuals.

## 2 Methods

### 2.1 Study Design

We conducted a MR study (Lawlor 2016) using genetic instruments derived from publicly available large‐scale GWAS to examine the association between AFSI and ISH and to assess potential mediating effects.

Our analysis comprised three stages:

Initially, we employed a two‐sample MR analysis to examine the total effect of AFSI on ISH.

Subsequently, we performed a two‐step MR analysis to investigate the potential mediating roles of MDD, SCZ, and ADHD. This involved assessing the causal impact of AFSI on these three psychiatric disorders and, in turn, their causal impact on ISH.

**FIGURE 1:**
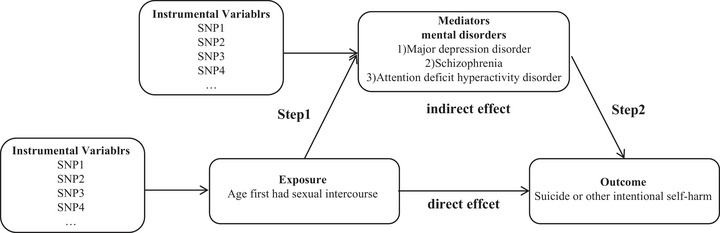
Flow of the MR study design.

Finally, we conducted a multivariable MR analysis to evaluate the direct effect of AFSI (the exposure) on ISH (the outcome) while accounting for the potential mediating factors (Figure 1).

### 2.2 Data Information

The data used in this study was sourced from the publicly available Open GWAS Project database (Hemani et al. 2018). Genetic predictors for AFSI were obtained from the largest GWAS conducted by the UK Biobank, using Phesant‐derived variables (output from the GWAS pipeline using Phesant‐derived variables from the UK Biobank) (Lu et al. 2023). Genetic predictors for ISH, whose population traits include suicide or other intentional self‐harm, were obtained from the most up‐to‐date GWAS conducted by the FinnGen Biobank, which included 52,208 cases and 166,584 controls. The genetic mediators for the three mental disorders were obtained from the GWAS datasets available from the Ieu Biobank, with sample sizes as follows: 59,851 cases and 113,154 controls for MDD, 52,017 cases and 75,889 controls for SCZ, and 20,183 cases and 35,191 controls for ADHD.

### 2.3 Selection of IVs

We performed a set of quality control steps to select suitable genetic instrumental tools (Chen et al. 2022). First, we obtained single‐nucleotide polymorphisms (SNPs) related to AFSI and three mental disorders (MDD, SCZ, and ADHD) (*p* < 5 × 10^−8^). Second, the LD between SNPs was eliminated because strong LD could lead to biased results (*r* ^2^ < 0.001, clumping distance = 10,000 kb). Third, we excluded SNPs strongly associated with self‐harm (*p* < 5 × 10^−8^). Moreover, 5e−8 was chosen as the *p* value threshold because it can effectively screen out SNPs that are significantly associated with exposure factors while maintaining a sufficient sample size for subsequent analysis. Third, we conducted SNP harmonization to correct the orientation of alleles. Fifth, to satisfy the strong association with exposure, we selected SNPs with *F* statistic > 10 as IVs. *F* statistics were calculated using the formula, $F=R^{2}\left(N-K-1\right)/\left(K(1-R^{2})\right)$, and *R* ^2^ was calculated using the formula (Burgess and Thompson 2011): $R^{2}=\left(2\times \mathrm{EAF}\times \left(1-\mathrm{EAF}\right)\times \beta^{2}\right)/((2\times \mathrm{EAF}\times \left(1-\mathrm{EAF}\right)\times \beta^{2})+$ $\left(2\times \mathrm{EAF}\times \left(1-\mathrm{EAF}\right)\times N\times SE^{2}\right))$.

### 2.4 Statistical Analysis

The main analysis utilized in this study was the random effect inverse variance weighted (IVW) method, chosen for its robustness and ability to provide a modest estimate even in the presence of heterogeneity (Palmer et al. 2011). In addition, we employed the weighted median (WM) and MR‐Egger (ME) methods to validate the robustness of IVW estimates. The WM estimate is determined by calculating the median of the weighted empirical distribution function of individual SNP ratio estimates. This approach yields a consistent effect estimate when more than 50% of the information is derived from valid SNPs (Bowden et al. 2016). The ME regression, on the other hand, provides a valid effect estimate even when all genetic variants are invalid instruments under the assumption that the association between each genetic variant and the exposure is independent of any pleiotropic effects (not influenced by the exposure itself) (Bowden, Davey Smith, and Burgess 2015). However, it is important to note that the WM and ME methods have reduced power compared to the IVW method, as reflected by wider confidence intervals (CIs) (Slob and Burgess 2020). Therefore, they serve as complementary methods in this study. Forest plots were employed to visually assess the results, treating an estimate from IVW, with the same direction as WM and ME, as a significant estimate. The details of the three main methods of MR analysis are introduced in the Supporting Information.

We also conducted several sensitivity analyses to ensure the robustness of our results. First, we employed the ME intercept test to detect the presence of horizontal pleiotropy, which occurs when the genetic variant affects the outcome through a pathway unrelated to the exposure. A significance level of *p* < 0.05 was used. Heterogeneity among different forms of inheritance was assessed using the Cochran Q statistic, with a significance level of *p* < 0.05. Furthermore, we performed a “leave‐one‐out” sensitivity analysis by sequentially excluding each SNP from the MR analysis. We identified and corrected outliers of pleiotropic biases using MR‐PRESSO for all reported results (Verbanck et al. 2018). The sensitivity analyses were visually represented using scatter and funnel plots.

Finally, we did mediation analysis using the product of the coefficients method and the difference method (Sanderson 2021; Zhao et al. 2022). The total effect (*β*) of AFSI on ISH was determined using univariable MR. The *β* was further decomposed into two components: (1) an indirect effect, which was obtained through a two‐step approach, where *β*_1_ represents the total effect of AFSI on MDD, and *β*_2_ represents the effect of MDD on ISH adjusting for AFSI, and the product method (*β*_1_ × *β*_2_). (2) A direct effect (*β* − *β*_1_ × *β*_2_). The same analytical process was applied to investigate other single mediators, such as SCZ and ADHD. When multiple mediators are involved, the indirect effect was derived using the difference method (*β* − *β*_3_), whereas *β*_3_ represents the MR effect of AFSI on ISH adjusted for genetically determined potential mediators. The proportion mediated was the indirect effect divided by the total effect.

Causal effects were reported as odds ratios (ORs) with corresponding 95% CIs and *p* values, with a significance level set at *p* < 0.05. The analysis was conducted using R software version 4.3.0, utilizing the TwoSampleMR and MR‐PRESSO packages. R reproduction codes are obtained in the Supporting Information.

## 3 Results

### 3.1 Selection of IVs

Genetic instruments for age first had sexual intercourse explained 2.2% of its variance, with an univariable *F*‐statistic of 45. The variance explained by, and *F*‐statistic for, genetic instruments for exposure were MDD 0.1% and 37, SCZ 5.5% and 45, and ADHD 0.6% and 34. The *F*‐statistics of all retained SNPs are over 10, indicating sufficient correlation strength between IVs and exposure. Thus, our study has no weak instrument bias.

### 3.2 Total Effect of Age First Had Sexual Intercourse on Suicide or Other Intentional Self‐Harm

**FIGURE 2:**
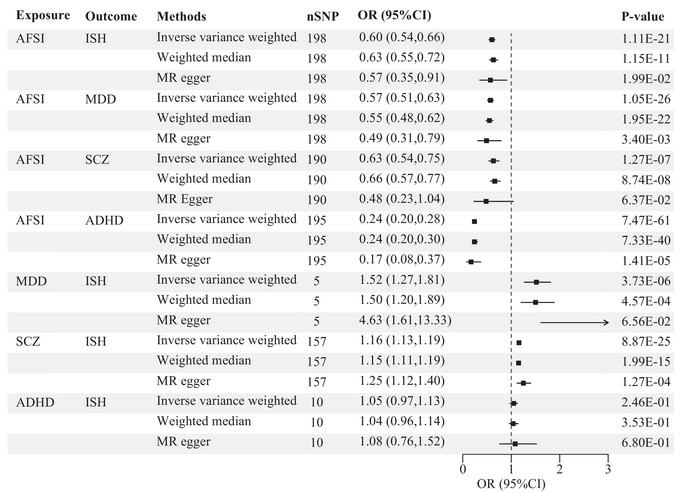
Univariate MR analysis of the effect of exposures on outcomes. ADHD, attention deficit hyperactivity disorder; AFSI, age first had sexual intercourse; ISH, intentional self‐harm; MDD, major depressive disorder; SCZ, schizophrenia; SNP, single‐nucleotide polymorphism.

Univariable MR analysis showed age at first had sexual intercourse was associated with higher suicide or other intentional self‐harm risk (IVW: OR = 0.60, 95% CI = 0.54–0.66, *p* < 0.05; Figure 2), and these findings were consistent with other methods used (Figure 2).

### 3.3 Effects of Age First Had Sexual Intercourse on the Risk of Mental Disorders

Univariable MR analysis showed age first had sexual intercourse was associated with higher MDD risk (IVW: OR = 0.57, 95% CI = 0.51–0.63, *p* < 0.05; Figure 2). Univariable MR analysis showed age first had sexual intercourse was associated with higher schizophrenia risk (IVW: OR = 0.63, 95% CI = 0.54–0.75, *p* < 0.05; Figure 2). The results were consistent with those of other methods except the ME. Univariable MR analysis showed age first had sexual intercourse was associated with higher ADHD risk (IVW: OR = 0.24, 95% CI = 0.20–0.28, *p* < 0.05; Figure 2).

### 3.4 Effects of Mental Disorders on the Risk of Suicide or Other Intentional Self‐Harm

Univariable MR analysis showed MDD was associated with suicide or other higher intentional self‐harm risk (IVW: OR = 1.52, 95% CI = 1.27–1.81, *p* < 0.05; Figure 2). Univariable MR analysis showed schizophrenia was associated with higher suicide or other intentional self‐harm risk (IVW: OR = 1.16, 95% CI = 1.13–1.19, *p* < 0.05; Figure 2).

### 3.5 Sensitivity Analyses

In addition, the estimates from IVW showed a significant and consistent relationship with both WE and ME in terms of magnitude and direction, suggesting that our results were not greatly biased by horizontal pleiotropy (Figure 2). Our study observed substantial heterogeneity with AFSI, MDD, and ADHD as the exposure (*p* > 0.05; Table 1), indicating potential pleiotropy of SNP effects. To evaluate potential directional horizontal pleiotropy, we performed ME regression by assessing whether the mean value of the Egger intercept was non‐zero, in which case pleiotropy could be directional (Bowden, Davey Smith, and Burgess 2015). Although there was heterogeneity, it did not affect the results of IVW, and our conclusions are reliable. In our study, we found no significant pleiotropy for any of the MR analyses (*p* > 0.05; Table 1). When removing outliers, MR PRESSO findings were consistent, and there was no pleiotropy bias (Table 2). The leave‐one‐out plots, scatter plots, and funnel plots all provided evidence of the robustness of our results (Figures S1–S3). Overall, our sensitivity analysis demonstrates the stability and robustness of our findings.

**TABLE 1:** Heterogeneity and pleiotropy analysis.

Exposure	Outcome	Heterogeneity			Pleiotropy	
		Method	Cochran's *Q*	*p* value	Egger intercept (95% CI)	*p* value
AFSI	ISH	IVW	340.70	9.07 × 10^−10^	1.67 (1.50, 1.86)	0.82
AFSI	MDD	IVW	350.94	9.38 × 10^−11^	0.57 (0.51, 0.63)	0.54
AFSI	SCZ	IVW	745.57	4.06 × 10^−67^	0.63 (0.54, 0.75)	0.48
AFSI	ADHD	IVW	343,16	2.21 × 10^−10^	1.01 (0.99, 1.02)	0.39
MDD	ISH	IVW	4.95	0.29	1.52 (1.27, 1.81)	0.13
SCZ	ISH	IVW	248.45	3.53 × 10^−6^	1.16 (1.13, 1.19)	0.17
ADHD	ISH	IVW	13.084	0.16	1.04 (0.64, 1.68)	0.87

Abbreviations: ADHD, attention deficit hyperactivity disorder; AFSI, age first had sexual intercourse; ISH, intentional self‐harm; IVW, inverse variance weighted.; MDD, major depressive disorder; SCZ, schizophrenia.

**TABLE 2:** MR‐PRESSO estimates between exposures and outcomes.

Exposure	Outcome	Raw estimates			Outlines corrected estimates		
		nSNP	*β*	*p* value	nSNP	*β*	*p* value
AFSI	ISH	198	−0.51	4.77 × 10^−18^	196	−0.49	1.53 × 10^−17^
AFSI	MDD	198	−0.56	2.38 × 10^−21^	197	−0.57	2.41 × 10^−22^
AFSI	SCZ	190	−0.46	3.47 × 10^−7^	173	−0.50	3.02 × 10^−19^
AFSI	ADHD	195	−1.45	1.15 × 10^−38^	193	−1.45	4.75 × 10^−40^
MDD	ISH	5	0.42	9.84 × 10^−3^	NA	NA	NA
SCZ	ISH	157	0.15	3.03 × 10^−19^	NA	NA	NA
ADHD	ISH	10	0.05	0.28	NA	NA	NA

Abbreviations: ADHD, attention deficit hyperactivity disorder; AFSI, age first had sexual intercourse; ISH, intentional self‐harm; MDD, major depressive disorder; SCZ, schizophrenia; SNP, single‐nucleotide polymorphism.

### 3.6 Mediated Effect and Proportion by MDD and SCZ

The multivariable MR analysis showed that the direct effects of AFSI on ISH were attenuated after adjusting for MDD (OR = 0.69, 95% CI = 0.61–0.79, *p* < 0.05; Table 3), SCZ (OR = 0.65, 95% CI = 0.63–0.67, *p* < 0.05; Table 3), and both MDD and SCZ (OR = 0.76, 95% CI = 0.67–0.86, *p *< 0.05; Table 3). The mediation analyses revealed that 31% of the association between AFSI and ISH was mediated through MDD, 12% through SCZ, and 46% through both MDD and SCZ (Table 3).

**TABLE 3:** Multivariate MR analysis of the direct effect of MDD and SCZ on ISH.

Exposure/outcome	Adjusted factors	Multivariate MR analysis			Mediation (%)
		nSNP	OR (95% CI)	*p* value	
AFIS/ISH	None	198	0.60 (0.54, 0.66)	1.11 × 10^−21^	
AFIS/ISH	MDD	191	0.69 (0.61, 0.79)	2.10 × 10^−8^	31
AFIS/ISH	SCZ	147	0.65 (0.63, 0.67)	1.13 × 10^−13^	12
AFIS/ISH	MDD, SCZ	147	0.76 (0.67, 0.86)	2.23 × 10^−5^	46

Abbreviations: ADHD, attention deficit hyperactivity disorder; AFSI, age first had sexual intercourse; ISH, intentional self‐harm; IVW, inverse variance weighted.; MDD, major depressive disorder; SCZ, schizophrenia.

## 4 Discussion

### 4.1 Introduction and Main Findings

To the best of our knowledge, our study represents the first investigation to establish a link between genetically predicted AFSI and an elevated risk of ISH. Previous observational studies have consistently demonstrated that early initiation of sexual activity, particularly among preteens, served as a significant predictor for the subsequent development of suicidal thoughts and suicide attempts in both genders (Kim and Kim 2010). The association between self‐harm and the pubertal stage was largely mediated by the onset of sexual activity (Patton et al. 2007). While previous research has primarily focused on suicide or self‐harm outcomes, our study provides compelling evidence regarding the relationship between AFSI and ISH. Observing the adverse outcomes of ESI from a genetic perspective, our study advocates for targeted interventions to delay sexual activities in adolescents, such as comprehensive adolescent sex education programs, to mitigate the risk of ISH.

### 4.2 Implications for Public Health and Clinical Practice

Our findings have significant implications for public health and clinical practice. The evidence supports the need for integrated screening protocols in adolescent health services to assess both sexual activity and mental health risk factors. Healthcare providers should receive enhanced training to recognize early sexual activity as a potential risk factor for future mental health issues and self‐harm. These findings underscore the need for comprehensive, age‐appropriate sexual education programs that not only provide information about safe sexual practices but also address the potential mental health implications of early sexual debut. Such programs could be integrated into school curricula and community health initiatives to reach a wider adolescent population (Patton et al. 2016; Salam et al. 2016).

### 4.3 ADHD and ISH: Unexpected Findings and Implications

Notably, no significant relationship was found between ADHD and ISH, contrary to some previous research (James, Lai, and Dahl 2004) or ADHD and smoking (Treur et al. 2021) or Omega 3–6 (Saeed et al. 2023); however, our study is the first MR to focus on the correlation between ADHD and the heightened risk of ISH. The absence of a robust causal relationship may imply potential sampling errors, highlighting the nuanced challenges inherent in cross‐database comparisons where exposure and outcome data are derived from distinct GWAS databases. Despite the observed deviation, it is crucial to note that ADHD still holds significance in our study, as it appears to heighten the risk of suicide by exacerbating the severity of comorbidities, notably conduct disorder and depression (Riglin et al. 2021). This intricate interplay between ADHD and its impact on associated conditions adds a layer of complexity to our understanding, emphasizing the need for further investigation and nuanced interpretation of the relationship between ADHD and ISH.

Although our study did not discover a significant connection between ADHD and ISH, it is important to note that ADHD still plays a significant role in the mental health of adolescents. The interaction between ADHD, comorbid conditions, and the risk of self‐harm is a complex issue that requires further investigation (Do et al. 2023). Specifically, longitudinal study designs should be utilized to examine the temporal dynamics of these relationships.

### 4.4 Mediation Analysis and Complex Relationship of Factors

Our mediation analysis further quantified the individual mediating effects, revealing that MDD and SCZ accounted for 31% and 12% of the mediation, respectively. Notably, when considered together, MDD and SCZ jointly mediated 46% of the observed effects. Building upon established knowledge, our findings align with previous studies that have highlighted the adverse consequences of early sexual activity (Tsuyuki et al. 2019), such as unwanted pregnancies (Louie et al. 2009), sexually transmitted diseases, and low self‐control (Magnusson, Crandall, and Evans 2019). These consequences, particularly linked to MDD and SCZ (Cai et al. 2022), underscore the intricate relationship between sexual behavior and mental health outcomes. The nuanced mediation proportions elucidate the varying contributions of distinct psychiatric factors, shedding light on the multifaceted nature of the association between AFSI and ISH. The association between early sexual activity and high‐risk factors such as childhood sexual abuse (Slavin et al. 2020) and forced sexual intercourse underscores the gravity of addressing adolescent ISH. Childhood sexual abuse emerges as a predisposing risk factor for suicide (Van Heeringen 2012) in individuals with SCZ, while SCZ (Roy 2005) and MDD contribute as potential stressors in hormonal models of suicidal behavior, amplifying the risk for self‐harm and suicide (Li et al. 2023). These factors have been identified as significant contributors to non‐suicidal self‐injury (Liu et al. 2021; Liu et al. 2018) and suicidal ideation (Pontes et al. 2022) among adolescents. Hence, a meticulous focus on enhancing (Lameiras‐Fernández et al. 2021) becomes imperative as a preventive measure to delay AFSI and mitigate the risk of ISH.

### 4.5 Potential Confounding Factors and Alternative Interpretations

Our findings warrant consideration of alternative explanations. Shared genetic factors might predispose individuals to both early sexual activity and increased self‐harm risk. Environmental factors, such as childhood adversity, could contribute to both early sexual debut and mental health issues. Personality traits like impulsivity may influence both AFSI and ISH (Mmari and Blum 2009). Reverse causality, where underlying mental health issues lead to both earlier sexual debut and increased ISH risk, is also possible. Cultural and societal factors may significantly mediate this relationship, potentially limiting generalizability. These confounding factors necessitate further investigation to strengthen the robustness of our conclusions.

### 4.6 Methodological Strengths

The present study boasts several methodological strengths that contribute to the robustness and reliability of our findings. First and foremost, the statistical data summary for exposure and outcomes derived from the largest and most recent GWAS (Uffelmann et al. 2021) ensures a comprehensive and up‐to‐date foundation for our analyses. Second, the exclusive utilization of genetic variants from individuals of European ancestry across all datasets serves to minimize potential population stratification bias, enhancing the generalizability and specificity of our results. A third strength lies in the establishment of strict criteria for the selection of IVs. This meticulous approach not only reflects methodological rigor but also bolsters the statistical power of our study, providing a more robust foundation for causal inference. Finally, to enhance the precision and reliability of our estimates, our research conducted sensitivity analyses utilizing multiple methods. This strategic approach serves to address issues such as horizontal pleiotropy and heterogeneity, fortifying the internal validity of our findings. On a broad spectrum, these methodological strengths collectively underscore the credibility and integrity of our study's outcomes.

### 4.7 Limitations and Considerations

Despite its strengths, our study has limitations. First, privacy constraints in the UK Biobank restrict access to crucial demographic information, prompting the need for gender and age stratification. Second, the exclusive use of European ancestry data limits global generalizability. This limitation is particularly significant given the potential global implications of these findings. Cultural differences in attitudes towards sexuality, mental health stigma, and healthcare access could substantially influence the AFSI‐ISH relationship in non‐European populations. Third, the study lacks a thorough assessment of intermediate factors, requiring additional analyses. Finally, the use of GWAS data introduces potential heterogeneities, preventing stratification analysis by age or gender. Finally, the use of GWAS data is powerful but it has limitations. It can introduce biases because it cannot account for gene‐environment interactions and epigenetic factors. To gain a more comprehensive understanding of the AFSI–ISH relationship, we need to supplement GWAS data with other research approaches. Twin studies and prospective cohort studies are examples of these complementary approaches.

### 4.8 Future Research Directions

Our findings underscore the critical need for targeted research to address the complex relationship between early sexual initiation and ISH. Given the escalating global prevalence of ESI among adolescents (Cerniglia and Cimino 2023) and the lack of scalable, effective guidance for adolescent sex education, developing optimal secondary prevention strategies is imperative (Mullins et al. 2014). We propose conducting randomized controlled trials to assess the impact of screening and treating MDD and SCZ on ISH risk in individuals with early sexual debut. Developing and evaluating comprehensive sexual education programs integrating mental health components is crucial. Future studies should investigate causal mechanisms linking early sexual initiation to ISH using advanced epidemiological methods. Longitudinal research examining long‐term mental health outcomes and ISH risk following early sexual debut is also warranted. These directions aim to inform evidence‐based interventions, addressing the urgent need for effective strategies given the increasing prevalence of ESI among adolescents globally.

## 5 Conclusion

This study establishes a causal link between younger AFSI and ISH, partially mediated by the impact on MDD and SCZ. Our results underscore the necessity for interventional studies to assess the efficacy of screening and treating MDD and SCZ in individuals with early sexual initiation. Such interventions may prove effective in mitigating the heightened risk of ISH associated with earlier sexual initiation compared to those with later initiation. The global implications of these findings emphasize the urgent need for culturally sensitive research and interventions addressing the complex interplay between sexual behavior, mental health, and self‐harm risk across diverse populations.

## Author Contributions

**Dameng Dong**: writing–original draft, writing–review and editing, conceptualization, investigation, visualization, data curation, validation. **Saboor Saeed**: writing–original draft, writing–review and editing, methodology, formal analysis. **Rucheng Chen**: methodology, writing–original draft, software. **Weijun Zheng**: writing–original draft, conceptualization, project administration. **An Chen**: writing–original draft, conceptualization, resources, supervision, funding acquisition.

## Ethics Statement

This study used publicly available data and did not require ethics approval. Ethical details can be found in the original publications of the contributing studies.

## Conflicts of Interest

The authors declare no conflicts of interest.

### Peer Review

The peer review history for this article is available at https://publons.com/publon/10.1002/brb3.70124.

## Supporting information



Supplementary Tables

Sup.F.1 The scatter plots of SNP effect of exposures on outcomes.
Sup.F.2 The funnel plots of SNP effect of exposures on outcomes.
Sup.F. 3 The leave‐one‐out plots of causal estimate.

Supplementary method material

## Data Availability

Our data is publicly available from genome‐wide association studies.
