# Supplementary material for: Investigating the Mediating Role of Mental Disorders in the Relationship Between Early Sexual Intercourse and Intentional Self‐Harm: A Two‐Step and Multivariable Mendelian Randomization Study
Source: Brain Behav. 2024 Dec 3;14(12):e70124. doi: 10.1002/brb3.70124 (PMC11615087; doi:10.1002/brb3.70124)
Supplement: Supplementary file 2 — Sup.F.1 The scatter plots of SNP effect of exposures on outcomes. Sup.F.2 The funnel plots of SNP effect of exposures on outcomes. Sup.F. 3 The leave‐one‐out plots of causal estimate. [file BRB3-14-e70124-s002.docx]

**Sup.F.1** The scatter plots of SNP effect of exposures on outcomes.

AFSI, age first had sexual intercourse; ISH, intentional self-harm; MDD, major depressive disorder; SCZ, schizophrenia; ADHD, attention deficit hyperactivity disorder; IVW, inverse variance weighted; SNP, single nucleotide polymorphism.

**Sup.F.2** The funnel plots of SNP effect of exposures on outcomes.

AFSI, age first had sexual intercourse; ISH, intentional self-harm; MDD, major depressive disorder; SCZ, schizophrenia; ADHD, attention deficit hyperactivity disorder; IVW, inverse variance weighted; SNP, single nucleotide polymorphism.

**Sup.F. 3** The leave-one-out plots of causal estimate.

AFSI, age first had sexual intercourse; ISH, intentional self-harm; MDD, major depressive disorder; SCZ, schizophrenia; ADHD, attention deficit hyperactivity disorder; IVW, inverse variance weighted; SNP, single nucleotide polymorphism.
