# Supplementary material for: Investigating the Mediating Role of Mental Disorders in the Relationship Between Early Sexual Intercourse and Intentional Self‐Harm: A Two‐Step and Multivariable Mendelian Randomization Study
Source: Brain Behav. 2024 Dec 3;14(12):e70124. doi: 10.1002/brb3.70124 (PMC11615087; doi:10.1002/brb3.70124)
Supplement: Supplementary file 3 — Supplementary method material [file BRB3-14-e70124-s001.docx]

# Supplementary method material

## R code

#### source of databases

**The source of these databases id can be found on the website “https://gwas.mrcieu.ac.uk”.**

**#age first had sexual intercourse ”ukb-b-6591”**

**#suicide or other inentional srlf-harm**

**“finn-b-VWXY20_SUICI_OTHER_INTENTI_SELF_H”**

**#major depression disorder “ieu-a-1188”**

**#schizophrenia “ieu-b-5102”**

**#ADHD “ieu-a-1183”**

#### R code reproduction process

**#Download and run the installation package**

**install.packages("TwoSampleMR")**

**library(TwoSampleMR)**

**install.packages("MR-PRESSO")**

**library(“MR-PRESSO”)**

**#Two sample MR analysis**

**#Repeat seven times for seven scenarios**

**exp<- extract_instruments(outcomes ="ukb-b-6591")**

**out <- extract_outcome_data(snps = exp$SNP, outcomes =**

**"finn-b-VWXY20_SUICI_OTHER_INTENTI_SELF_H")**

**out1 <- subset(out,out$pval.outcome>=5e-08)**

**dat<-harmonise_data(exposure_dat = exp, outcome_dat = out1,action = 1)**

**res<-mr(dat,method_list = c("mr_ivw","mr_egger_regression","mr_weighted_median"))**

**res**

**#sensitivity analyses**

**#MR-PRESSO**

**mr_presso(BetaOutcome = "beta.outcome", BetaExposure = "beta.exposure", SdOutcome = "se.outcome", SdExposure = "se.exposure", OUTLIERtest = TRUE, DISTORTIONtest = TRUE, data = as.data.frame(dat), NbDistribution = 1000, SignifThreshold = 0.05)**

**#**Heterogeneity analysis

**mr_heterogeneity(dat)**

**#**Pleiotropy analysis

**mr_pleiotropy_test(dat)**

**#MR-Egger intercept test**

**mr_egger_regression(b_exp =dat$beta.exposure, b_out = dat$beta.outcome, se_exp = dat$se.exposure,se_out =dat$se.outcome)**

**#leave-one-out test**

**mr_leaveoneout_plot(leaveoneout_results = mr_leaveoneout(dat))**

**#scater plot**

**mr_scatter_plot(mr_results = mr(dat,method_list = c("mr_ivw","mr_egger_regression","mr_weighted_median")),dat)**

**#funnel plot**

**mr_funnel_plot(singlesnp_results = mr_singlesnp(dat))**

**#Multivariable MR analysis**

**#We need to execute three times possible exposure combination.**

**mvmr<-mv_extract_exposures(c("ukb-b-6591","ieu-a-1188"))**

**mvmr<-mv_extract_exposures(c("ukb-b-6591",“ieu-b-5102”))**

**mvmr<-mv_extract_exposures(c("ukb-b-6591","ieu-a-1188"，“ieu-b-5102”))**

**mvmr_outcome_dat<-extract_outcome_data(mvmr$SNP, "finn-b-VWXY20_SUICI_OTHER_INTENTI_SELF_H")**

**mvmr_outcome_dat1 <- subset(mvmr_outcome_dat,mvmr_outcome_dat$pval.outcome>=5e-08)**

**mvmr_dat<-mv_harmonise_data(mvmr, mvmr_outcome_dat1,harmonise_strictness =1 )**

**mvmr_res<-mv_multiple(mvmr_dat)**

**mvmr_res**

### Details of three two-sample MR methods

In Inverse Variance Weighted (IVW), which we consider to be the primary analysis (with other methods providing significant sensitivity analyses), we combined the SNP-specific Wald ratio estimates using the IVW. However, it is important to note that this method may introduce bias if any of the instrumental variables are invalid. In other words, if these variables influence the outcome through pathways independent from the exposure of interest, known as horizontal pleiotropy [1].

To account for this potential bias, we also employed two additional methods: weighted median and MR-Egger [2]. These methods aim to obtain a reliable MR estimate that is robust against horizontal pleiotropy. Each method operates under different assumptions, which are described below.

#### Inverse Variance Weighted (IVW)

The IVW method combines the SNP-specific Wald estimates, which represent the ratio of the SNP's effect on the outcome to its effect on the exposure. The formulas used are as follows:


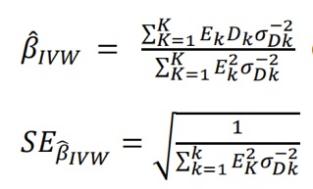


where $E_{k}$ is the mean change in the exposure level per additional effect allele of SNP k, $D_{K}$ is the mean change in outcomes per additional effect allele of SNP k with standard error $\sigma_{D_{K}}$.

The results obtained using the IVW method were also utilized to estimate the mental disorders on the causal relationship between exposure and outcome, for example, between age first had sexual intercourse and suicide or other Intentional self-harm.

This method provides a consistent estimate of the causal effect if all genetic variants (SNPs) used as instrumental variables satisfy the instrumental variable (IV) assumptions. Specifically, these assumptions are: 1) the genetic variants predict the exposure, 2) the genetic variants are independent of any confounding factors related to the association between the exposure and outcome, and 3) the genetic variants are independent of the outcome given the exposure and confounding factors (exclusion restriction criteria). However, this method does not test for or consider horizontal pleiotropy, which, if present, would violate the exclusion restriction criteria assumption and potentially bias the estimate of the causal effect. The IVW estimate is a statistically efficient method but can still be biased if even one genetic variant is invalid, meaning it has horizontal pleiotropic effects. To account for the possibility of the presence of invalid genetic instrumental variables, we also incorporated the weighted median method in our analysis.

#### Weighted median estimator

The weighted median estimator is an enhanced version of the simple median approach that takes into account the variance of individual genetic instruments. Unlike the simple median, the weighted median estimator calculates the median of a distribution with estimate $\beta^{j}$ as its $P_{j}=100{(S_{j}-W_{j}/2)}^{th}$ percenstile. Here, P represents the percentile for the $j^{th}$ ordered ratio estimate, $W_{j}$ is the weight assigned to the $j^{th}$ ordered ratio estimate, which is proportional to the inverse of the instrumental variable (IV) variance, and $S_{j}$ is the cumulative sum of weights up to and including the weight of the jth ordered ratio estimate, calculated using Equation: $S_{j}=\sum_{K=1}^{j} W_{k}$.

The weights are standardized so that the sum of the weights Sj equals one. Similar to the simple median approach, the weighted median estimator assumes that no more than 50% of the genetic instruments are invalid. Additionally, it assumes that no individual IV contributes more than 50% of the total weight. Compared to the simple median method, the weighted median method is more statistically efficient.

#### MR-Egger regression

The MR-Egger method was developed by Bowden et al. as a specific approach to test for horizontal pleiotropy and to correct for it in Mendelian randomization (MR) analyses [1]. MR-Egger utilizes a weighted linear regression of the gene-outcome coefficients θj on the gene-exposure coefficients$\delta_{j}$: $\theta_{j}$= $\beta_{OE}$ + $\beta_{E}*\delta_{j}$. In this regression, all the $\delta_{j}$ associations are oriented to be positive, and the weights are the inverse variances of the gene-outcome associations ($\sigma_{Y_{j}}^{-2}$).

If the intercept in the MR-Egger regression model were truly zero (or constrained to be zero), the MR-Egger slope estimate $\beta_{E}$ would be the same as the β estimate from the inverse-variance weighted (IVW) method. A zero intercept ($\beta_{OE}$) suggests no violation of the exclusion restriction criteria, indicating no horizontal pleiotropy. It provides an estimate of the average pleiotropic effect across all genetic variants, reflecting the joint influence of the instruments on the outcome in the absence of any effect of the genetic variants on the risk factor.

A non-zero intercept suggests the presence of horizontal pleiotropy, indicating potential bias in the IVW estimate. Moreover, the MR-Egger method yields consistent estimates (via the MR-Egger slope) for the true causal effect even if all genetic variants suffer from invalidity due to horizontal pleiotropy. This means that it represents the causal effect in MR analysis while accounting for violations of the exclusion restriction criteria. However, it relies on an additional assumption known as the InSIDE (instrument strength independent of direct effect) assumption, which requires that the genetic effects on the outcome operate directly rather than through confounders. If the pleiotropic effects of genetic variants are mediated solely through a single confounder, they will be correlated with instrument strength, thereby violating the InSIDE assumption.

**Reference**

1. Bowden, J., et al., *Consistent Estimation in Mendelian Randomization with Some Invalid Instruments Using a Weighted Median Estimator.* Genet Epidemiol, 2016. **40**(4): p. 304-14.

2. Bowden, J., G. Davey Smith, and S. Burgess, *Mendelian randomization with invalid instruments: effect estimation and bias detection through Egger regression.* Int J Epidemiol, 2015. **44**(2): p. 512-25.
